# Long term liver specific glucokinase gene defect induced diabetic cardiomyopathy by up regulating NADPH oxidase and down regulating insulin receptor and p-AMPK

**DOI:** 10.1186/1475-2840-13-24

**Published:** 2014-01-22

**Authors:** Hui Li, Xi Wang, Yiqing Mao, Ruobi Hu, Wei Xu, Zhen Lei, Na Zhou, Ling Jin, Tingting Guo, Zhixin Li, David M Irwin, Gang Niu, Huanran Tan

**Affiliations:** 1 Department of Pharmacology, Health Science Center, Peking University, 38 Xue Yuan Road, Beijing 100191, China; 2 Beijing N&N Genetech Company, Beijing, China; 3 Department of Laboratory Medicine and Pathobiology, University of Toronto, Toronto, Ontario M5S 1A8, Canada; 4 Department of Pharmacology, Ningxia Medical University, Yinchuan 750004, China; 5 Department of the Integration of Chinese and Western Medicine, Health Science Center, Peking University, Beijing 100191, China

**Keywords:** Liver-specific glucokinase knockout, Diabetic cardiomyopathy, Rosiglitazone, Insulin receptor, AMPK

## Abstract

**Background:**

The liver-specific glucokinase knockout (gck^w/–^) mouse experiences long-term hyperglycemia and insulin resistance. This study was designed to evaluate the functional and structural changes in the myocardium of 60 week-old gck^w/–^ mice, and to investigate the effect of rosiglitazone on the myocardium in this model.

**Methods:**

60 week-old gck^w/–^ mice were randomly divided into 3 groups: gck^w/–^, gck^w/–^ mice treated with insulin (1 U/kg) and gck^w/–^ mice treated with rosiglitazone (18 mg/kg). Insulin or rosiglitazone treatment was for 4 weeks. Gck^w/w^ litermates were used as controls. Echocardiography, electrocardiogram, biochemical, histopathological, ultrastructural, real time PCR and Western blot studies were performed to examine for structural and functional changes.

**Results:**

Long-term liver-specific gck knockout in mice elicits hyperglycaemia and insulin resistance. Compared to age matched gck^w/w^ mice, 60 week-old gck^w/–^ mice showed decreased LV internal dimension, increased posterior wall thickness, lengthened PR and QRS intervals, up-regulated MLC2 protein expression, decreased SOD activity, increased MDA levels and up-regulated Cyba mRNA. Morphological studies revealed that there was an increase in the amount of PAS and Masson positively stained material, as did the number and proportion of the cell occupied by mitochondria in the gck^w/–^ mice. Western blot analysis revealed that the levels of the insulin receptor, Akt, phosphorylated AMPK beta and phosphorylated ACC were reduced in gck^w/–^ mice. These effects were partly attenuated or ablated by treatment with rosiglitazone.

**Conclusions:**

Our results indicate that changes in the myocardium occur in the liver-specific glucokinase knockout mouse and suggest that reduced glucokinase expression in the liver may induce diabetic cardiomyopathy by up regulating NADPH oxidase and down regulating insulin receptor and p-AMPK protein levels. Rosiglitazone treatment may protect against diabetic cardiomyopathy by altering the levels of a set of proteins involved in cardiac damage.

## Background

Diabetic cardiomyopathy (DCM) is defined as structural and functional changes in the myocardium, which are independent of hypertension, chronic artery disease or any other known cardiac diseases, and are caused by metabolic and cellular abnormalities induced by diabetes mellitus (DM). One of the most important structural hallmarks of DCM is cardiac hypertrophy [1,2]. Hyperglycemia has been viewed as the pivotal pathogenetic factor for the development of DCM. In fact, it can cause abnormalities at the cardiac myocyte level, eventually leading to functional and structural abnormalities, including systolic and diastolic dysfunction, as well as cardiac hypertrophy and myocardial fibrosis [3]. However, other factors seem to be involved in the evolution of the disease, including hyperinsulinemia, insulin resistance, oxidative stress, inflammation, endothelial dysfunction and apoptosis [1,2,4]. Animal models have been used to study the mechanisms underlying DCM [5-8]. In some animal models of DCM, drug treatment, or the effects of genetic mutation often leads to obesity and diabetes. All of these models have limitations and none are a perfect phenocopy of the human condition [9].

Maturity-onset diabetes of the young (MODY) is a clinically heterogeneous group of disorders and accounts for about 2%–5% of all diabetic patients [10]. To date, five proteins have been identified whose genetic absence or impairment causes MODY, the enzyme glucokinase (GCK/MODY2) and four transcription factors: hepatocyte nuclear factor (HNF) 4α/MODY1, HNF-1α/MODY3, insulin promoter factor 1/MODY4 and HNF-1β/MODY5. MODY2 is one of the most prevalent subtypes of MODY and is associated with mutations in the glucokinase gene on Chromosome 7p and is characterized by chronic mild hyperglycemia, with an onset usually before the age of 25 [11,12]. Little data is available on MODY2, as only a few mouse models have been developed [10,13,14]. Bali et al. [12] used homologous recombination in mouse embryonic stem cells to assess the effects of disrupting GCK function in both β-cells and hepatocytes as an animal model for MODY2. A liver-specific glucokinase knockout mouse has been constructed as a model for the liver-specific role of glucokinase in MODY2, independent of its function in regulating insulin secretion in pancreatic β-cells. Liver-specific glucokinase knockout mice were generated using the Cre-loxP gene targeting strategy, and the primary effects of the deletion of the gene have been evaluated [15].

The nuclear transcription factor peroxisome proliferator activated receptor γ (PPARγ) is a key regulator in adipogenesis. Thiazolidinediones, agonists of PPARγ, are an established and effective treatment for patients with type 2 diabetes [16]. Recently, there has been some controversy regarding an increase in cardiovascular events, including myocardial infarction and congestive heart failure, in humans with the use of the PPARγ agonist, rosiglitazone [17,18].

The aim of this study was to investigate the functional and structural changes in the myocardium that result from long-term reduced liver gck expression in our mouse liver-specific gck^w/–^ MODY2 model and to determine whether rosiglitazone or insulin can reverse these changes in these mice.

## Methods

### Animals

A protocol for these experiments, following the “Guidelines for Animal Experiments”, was approved by the Peking University Health Science Center. Liver-specific gck knockout mice were previously generated by our lab [15]. Liver-specific gck knockout mice (gck^w/–^) were randomly divided into 3 groups: untreated (gck^w/–^, n = 9), gck^w/–^ treated with insulin (gck^w/–^ + Ins, n = 9) and gck^w/–^ treated with rosiglitazone (gck^w/–^ + RSG, n = 9). Wild-type littermates (gck^w/w^, n = 9) were used as controls. Insulin (1 U/kg/day) was administrated to the gck^w/–^ mice by subcutaneous injection for 4 weeks (gck^w/–^ + Ins). Rosiglitazone (18 mg/kg/day) was administrated to the gck^w/–^ mice by gavage for 4 weeks (gck^w/–^ + RSG).

### Assay of biochemistry parameters

An intraperitoneal glucose tolerance test (ipGTT) was performed in each group by intraperitoneal injection of a 20% glucose solution at a dose of 2 g/kg. Tail vein blood glucose levels were measured using a Roche blood glucose monitor (Glucotrend 2, Roche, Germany) in samples taken immediately before the glucose injection and at 30, 60, and 120 minutes after. Fasting insulin levels were quantified using a commercially available radioimmunoassay kit (China Institute of Atomic Energy, Beijing, China). Insulin sensitivity and β-cell capability of individual animals was evaluated using the homeostasis model assessment (HOMA) index [19]. The formula used was the following:

$$\begin{array}{l} \left[\text{HOMA}-\text{IR}\right]=\text{fasting}\;\text{serum}\;\text{glucose}\left(\text{mmol}/L\right)\\ \;\times \text{fasting}\;\text{serum}\;\text{insulin}\left(\text{mIU}/L\right)/22.5.\\ \left[\text{HOMA}-\beta -\mathrm{cell}\right]=20\times \text{fasting}\;\text{serum}\;\text{insulin}\left(\text{mIU}/L\right)/\\ \;\left(\text{fasting}\;\text{serum}\;\text{glucose}\left(\text{mmol}/L\right)-3.5\right). \end{array}$$

### Echocardiography

Transthoracic echocardiography was performed on pentobarbital anesthetized mice at rest using a high-resolution imaging system for small animals (Vevo 770, VisualSonics, Toronto, Canada), equipped with a high-frequent ultrasound probe (RMV-707B). Parasternal long-axis and short-axis views were acquired. Left ventricular (LV) dimensions and wall thicknesses were determined from parasternal short axis M-mode images. Ejection fraction (EF) and fractional shortening (FS) were calculated using the Vevo770 software. The final data represent the averaged values of 3–6 cardiac cycles.

### Electrocardiographic recordings

Surface electrocardiographic (ECG) recordings were obtained from conscious mice. For analysis of ECGs, the onsets and offsets of the P, Q, R, S, and T waves were determined by measuring the earliest (onset) and the latest (offset) times from lead II.

### Western blot

Sodium dodecyl sulfate-polyacrylamidegel electrophoresis (SDS-PAGE) and Western blot to measure myocardium myosin light chain (MLC2), insulin receptor β (IRβ), Akt, adenosine 5′-monophosphate (AMP)-activated protein kinase alpha (AMPKα), p-AMPKα, AMPKβ1/2, p-AMPKβ1, acetyl-CoA carboxylase (ACC) and p-ACC were performed. Heart tissue was homogenized and protein was quantified using the Bradford method. Sixty micrograms of protein was separated on SDS-PAGE. Separated proteins were transferred by electroelution onto PVDF paper (Millipore, Billerica, USA). Blots were probed with rabbit antibodies to insulin receptor β, Akt, AMPKα, Phospho-AMPKα (Thr172), AMPKβ1/2, Phospho-AMPKβ1 (Ser108), ACC and Phospho-ACC (Ser79). All antibodies were obtained from Cell Signaling Technology (Beverly, MA, USA) and used at 1:1000 dilution with incubation overnight at 4°C. Anti-mouse MLC2 antibody was procured from Santa Cruz Biotechnology (Dallas, USA). Antibody binding was detected after incubation with appropriate secondary antibodies conjugated with HRP, with the membrane-bound antibodies visualized by luminal chemiluminescence ChemiDoc XRS (Bio-Rad, Hercules, CA, USA).

### Reverse transcription and real-time PCR

Heart tissues were homogenized and total RNA was isolated using Trizol LS reagent (Invitrogen, Carlsbad, USA) according to the manufacturer’s instruction. Amplifications were performed with the BIO-RAD Miniopticon TM Real-Time PCR Detection system CFB-3120 using iQTM SYBR Green Supermix 170–8880 (Bio-Rad) with the primers listed in Table 1. Amplifications were performed using the following conditions: initial denaturation at 95°C for 10 min followed by 39 cycles performed at 95°C for 15 s and 67°C for 1 min. Transcription levels were normalized to those of beta actin.

**Table 1:** List of primers used in this study

	**Forward primer**	**Reverse primer**
Cyba	CATGTGGGCCAACGAACAG	CACTGTGTGAAACGTCCAGCAGTA
Cybb	TGATCCTGCTGCCAGTGTGTC	GTGAGGTTCCTGTCCAGTTGTCTTC
Fibronectin 1	GCTTTGGCAGTGGTCATTTCAG	ATTCCCGAGGCATGTGCAG

### PAS staining

Prepared heart slides were stained with periodic acid-Schiff’s reagent (PAS) for glycogen and evaluated by light microscopy. The glycogen content of the myocardial tissue sections was expressed as the relative area of positive PAS-stained tissue (purple red-stained glycogen) to the total tissue area using Image-Pro Plus 6 (Media Cybernetics, Rockville, USA).

### Masson staining

Mouse cardiac tissue was first fixed on Bouin’s fixative and stained with Weigert’s hematoxylin and Biebrich scarlet-acid fuchsin (Sigma, Milwaukee, USA), followed by treatment with phosphomolybdic–phosphotungstic acid solution (Sigma) and staining by methyl blue and Mayer’s Hematoxylin and Eosin. The extent of fibrosis in the myocardial tissue sections was quantified using Image-Pro Plus 6 (Media Cybernetics, Rockville, USA) as the relative area of positive stained area (blue–green fibrosis) normalized to the total tissue area.

### Electron microscopy

Samples were collected from freshly excised left ventricular myocardium and processed as described previously [20]. The proportion of the cell volume occupied by mitochondrial (volume density) and the number of mitochondria were analyzed in a blinded fashion by the point counting method using Image-Pro Plus 6 (Media Cybernetics, Rockville, USA). Mitochondrial volume density was quantified as the relative area of mitochondrial area normalized to the total cell area.

### SOD activity and MDA levels

Superoxide dismutase (SOD) activities and malondialdehyde (MDA) levels in the myocardial tissues and serum were determined using commercially available kits (Nanjing Jiancheng Bioengineering Institute, Nanjing, China).

### Statistical analysis

Results are shown as means ± SD. Differences between the control and experimental groups were evaluated by one-way (ANOVA; SPSS 13.0 for Windows, SPSS Inc., Chicago, IL, USA). P values less than 0.05 were considered to be statistically significant.

## Results

### Effects of the liver-specific gck gene knockout on glucose homeostasis and insulin resistance in mice

**Figure 1:**
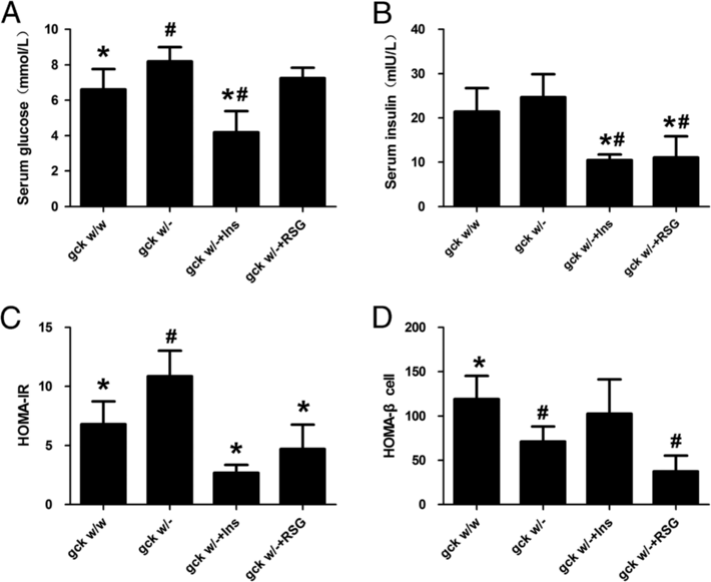
**Effect of rosigitizone and insulin on fasting glucose (A), insulin (B), HOMA-IR (C) and HOMA-β-cell (D) levels in 60-week old gck**^**w/– **^**mice.** Glucose and insulin levels as well as calculated HOMA-IR and HOMA-β-cell levels are shown for 60-week old wild-type (gck^w/w^) and liver-specific gck knockout (gck^w/–^) mice as well as gck knockout mice treated with insulin or rosigitizone for 4 weeks. n = 6 for all samples. Asterisk (*) refers to statistical significance (P < 0.05) in comparisons with gck^w/–^ mice, while #refers to comparisons with gck^w/w^ mice.

**Figure 2:**
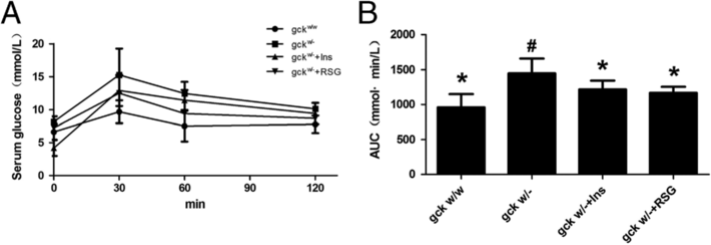
**Intra-peritoneal glucose tolerance tests (ipGTT) in 60-week old liver-specific gck knockout mice.** ipGTT **(A)** and areas under the curve of the ipGTT (AUC)** (B)** are shown. Glucose levels were measured at 0, 30, 60, 120 minutes after glucose injection in wild-type (gck^w/w^) and gck knockout (gck^w/–^) mice as well as knockout mice treated with insulin or rosiglitazone for 4 weeks. n = 6 for all samples. Asterisk (*) refers to statistical significance (P < 0.05) in comparisons with gck^w/–^ mice, while # refers to comparisons with gck^w/w^ mice.

Fasting glucose and HOMA-IR levels were significantly higher and HOMA-β-cell levels significantly lower in liver-specific gck knockout mice (gck^w/–^) than in gck^w/w^ mice (Figure 1). In the gck^w/–^, treatment with rosiglitazone did not change the fasting glucose and calculated HOMA-β-cell levels, but did result in a significant decrease in both the fasting insulin and calculated HOMA-IR levels (p < 0.05, Figure 1). Glucose levels at 0, 30, 60, and 120 minutes after glucose injection and the AUC were significantly higher in the gck^w/–^ than in the gck^w/w^ mice (p < 0.05) (Figure 2). Compared to the pre-treatment responses, rosiglitazone treatment decreased the AUC and the impairment in the glucose tolerance response in the gck^w/–^ mice, but only reached significance at the 60 and 120-minute time points after glucose injection (p < 0.05) (Figure 2).

### Left ventricle internal dimension and posterior wall thickness is deteriorated in the liver-specific gck gene knockout mouse

Doppler and M-mode images revealed that significant echocardiographic changes are found in the gck^w/–^ mice. Left ventricle (LV) internal dimension during diastole (LVID;d) and systole (LVID;s) were significantly decreased in the gck^w/–^ mice, compared to gck^w/w^ mice. LVID;d significantly increased after treatment with insulin or rosiglitazone. LV posterior wall thickness during diastole (LVPW;d) and systole (LVPW;s) were significantly increased in the gck^w/–^ mice, compared to gck^w/w^ mice. Changes in the ejection fraction (%EF) and fraction of shortening (%FS) were not statistically different in the gck^w/–^ mice, compared to gck^w/w^ mice, however insulin or rosiglitazone treatment yielded significant increases (p < 0.05,versus gck^w/–^) (Table 2).

**Table 2:** **Echocardiographic measurements (M-mode) in gck**^**w/– **^**and gck**^**w/w **^**mice (n = 3)**

	**gck**^**w/w**^	**gck**^**w/–**^	**gck**^**w/–**^ **+ Ins**	**gck**^**w/–**^ **+ RSG**
LVID;d	2.55 ± 0.15^*^	2.06 ± 0.16^#^	2.54 ± 0.15^*^	2.50 ± 0.12^*^
LVPW;d	0.84 ± 0.10^*^	1.03 ± 0.06^#^	1.27 ± 0.10^#*^	1.08 ± 0.07^#^
LVID;s	1.15 ± 0.05^*^	0.84 ± 0.08^#^	0.86 ± 0.07^*^	0.89 ± 0.11^*^
LVPW;s	1.14 ± 0.14^*^	1.54 ± 0.17^#^	1.90 ± 0.09^#*^	1.74 ± 0.03^#*^
LVAW;d	0.93 ± 0.09	0.83 ± 0.21	1.19 ± 0.09^#*^	0.94 ± 0.06
LVAW;s	1.12 ± 0.42	1.48 ± 0.15	1.76 ± 0.05^*^	1.48 ± 0.39
%EF	86.87 ± 1.97	90.62 ± 2.31	94.08 ± 1.23^*^	93.23 ± 2.07^*^
%FS	54.49 ± 2.58	59.31 ± 3.52	66.19 ± 2.59^#*^	64.61 ± 4.01^#*^

Data are expressed as the mean ± standard. LVID;d, LV internal diameter in diastole. LVPW;d, LV posterior wall thickness in diastole. LVID;s, LV internal diameter in systole. LVPW;s, LV posterior wall thickness in systole. LVAW;d, LV anterior wall thickness in diastole. LVAW;s, LV anterior wall thickness in systole. EF, Ejection fraction. FS, Fractional shortening. n = 3 for all samples. Asterisk (*) refers to statistical significance (P < 0.05 for *) in comparisons with gck^w/–^ mice, while # refers to statistical significance (P < 0.05 for #) in comparisons with gck^w/w^ mice.

### PR and QRS intervals are lengthened in the liver-specific gck knockout mouse

Surface ECG recordings showed significantly longer PR intervals in the gck^w/–^ mice versus gck^w/w^ mice, as did QRS intervals (Table 3). Treatment of gck^w/–^ mice with insulin or rosiglitazone significantly shortened both the PR intervals and QRS intervals (p < 0.05, Table 3). Changes in heart rates, P Duration, QT intervals, and corrected QT (QTc) intervals were not statistically different among the four groups.

**Table 3:** **ECG parameters in gck **^**w/– **^**and gck**^**w/w **^**mice (n = 6)**

	**gck**^**w/w**^	**gck**^**w/–**^	**gck**^**w/–**^ **+ Ins**	**gck**^**w/–**^ **+ RSG**
Heart Rate(BPM)	613.9 ± 51.47	537.9 ± 65.6	592.6 ± 87.3	505.3 ± 39.9
P Duration(ms)	9.22 ± 1.64	14.82 ± 7.68	8.06 ± 1.96	10.82 ± 1.36
PR Interval(ms)	38.28 ± 2.31^*^	44.77 ± 7.46^#^	36.75 ± 6.29^*^	38.41 ± 0.67^*^
QRS Interval(ms)	8.54 ± 1.35^**^	13.35 ± 3.28^##^	8.37 ± 1.87^*^	9.77 ± 0.71^*^
QT Interval(ms)	18.06 ± 1.56	21.04 ± 2.28	16.98 ± 0.60	16.13 ± 1.89
QTc(ms)	58.10 ± 3.83	63.28 ± 10.73	53.36 ± 5.81	46.92 ± 6.97

Data are expressed as the mean ± standard. QTc, Corrected QT Interval. QTc = QT/(RR/100)1/2. n = 3 for all samples. Asterisk (*) refers to statistical significance (P < 0.05 for * and P < 0.005 for **) in comparisons with gck^w/–^ mice, while # refers to statistical significance (P < 0.05 for # and P < 0.005 for ##) in comparisons with gck^w/w^ mice.

### Liver-specific gck gene knockout mice have increased myocardium MLC2 protein levels

**Figure 3:**
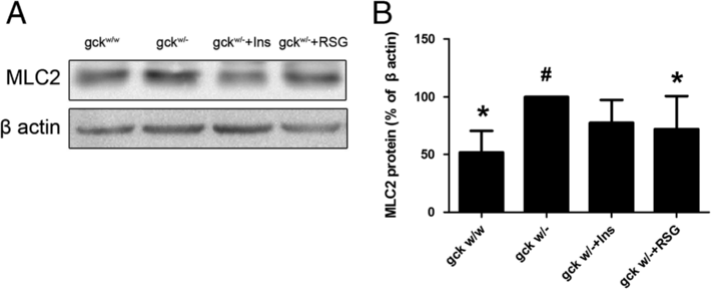
**Quantification of the protein levels of myosin light chain 2 (MLC2) in 60-week old liver-specific gck knockout mice.** Representative Western blot images **(A)** and quantification of the MLC2 Western blots in heart homogenates **(B)** are shown for wild-type (gck^w/w^) and gck knockout (gck^w/–^) mice as well as knockout mice treated with insulin or rosiglitazone for 4 weeks. n = 3 for all samples. Asterisk (*) refers to statistical significance (P < 0.05) in comparisons with gck^w/–^ mice, while # refers to comparisons with gck^w/w^ mice.

Myosin light chain 2 (MLC2) is expressed in the heart in a restricted manner during development and has been shown to play a key role in cardiogenesis [21]. We performed Western blot analysis to measure the protein levels of MLC2 and found that they were significantly increased in the gck^w/–^ mice, compared with the gck^w/w^ mice, and were restored to more normal levels with rosiglitazone or insulin treatment (p < 0.05, Figure 3).

### Liver-specific gck gene knockout aggravates oxidative stress in mouse serum and myocardium tissue

**Figure 4:**
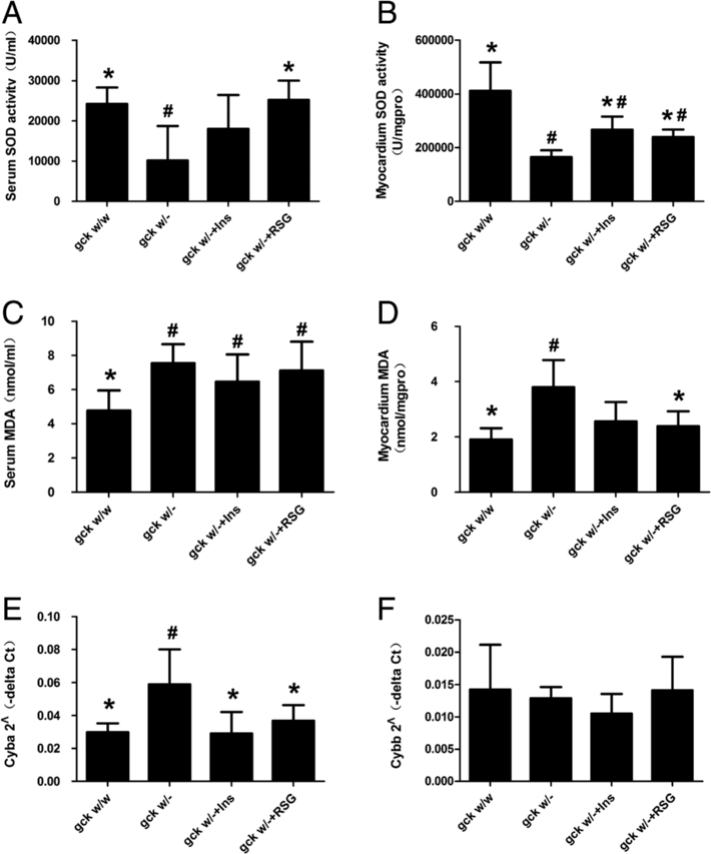
**Oxidative stress in 60-week old liver-specific gck knockout mice.** Serum and myocardium SOD activity **(A and B)**, MDA levels **(C and D)**, and relative levels of mRNA for Cyba **(E)** and Cybb **(F)** in the myocardial are shown for wild-type (gck^w/w^) and gck knockout (gck^w/–^) mice as well as knockout mice treated with insulin or rosiglitazone for 4 weeks. mRNA levels were determined by real-time PCR and corrected with beta actin as an internal standard. n = 6 for all samples. Asterisk (*) refers to statistical significance (P < 0.05) in comparisons with gck^w/–^ mice, while # refers to comparisons with gck^w/w^ mice.

Our data showed a significant reduction in SOD activity and an elevation of MDA levels in gck^w/–^ mice, compared to gck^w/w^ mice, in serum and myocardium tissue (Figure 4). Serum and myocardium SOD activities were significantly increased after treatment with rosiglitazone (p < 0.05), however only myocardium SOD activity was significantly increased after treatment with insulin (p < 0.05). Myocardium MDA levels were significantly decreased after treatment with rosiglitazone (p < 0.05), but not with insulin. There was no significant difference after treatment with rosiglitazone or insulin in serum MDA levels. As shown in Figure 4E, the level of cytochrome b-245 alpha (Cyba) mRNA in the myocardium of gck^w/–^ mice was significantly higher than that of gck^w/w^ mice, and was normalized by treatment with rosiglitazone or insulin (p < 0.05). Cytochrome b-245 beta (Cybb) mRNA levels did not show a significant change, whether untreated or when the mice were treated with rosiglitazone or insulin.

### Cardiac fibrosis was increased in the liver-specific gck knockout mouse

**Figure 5:**
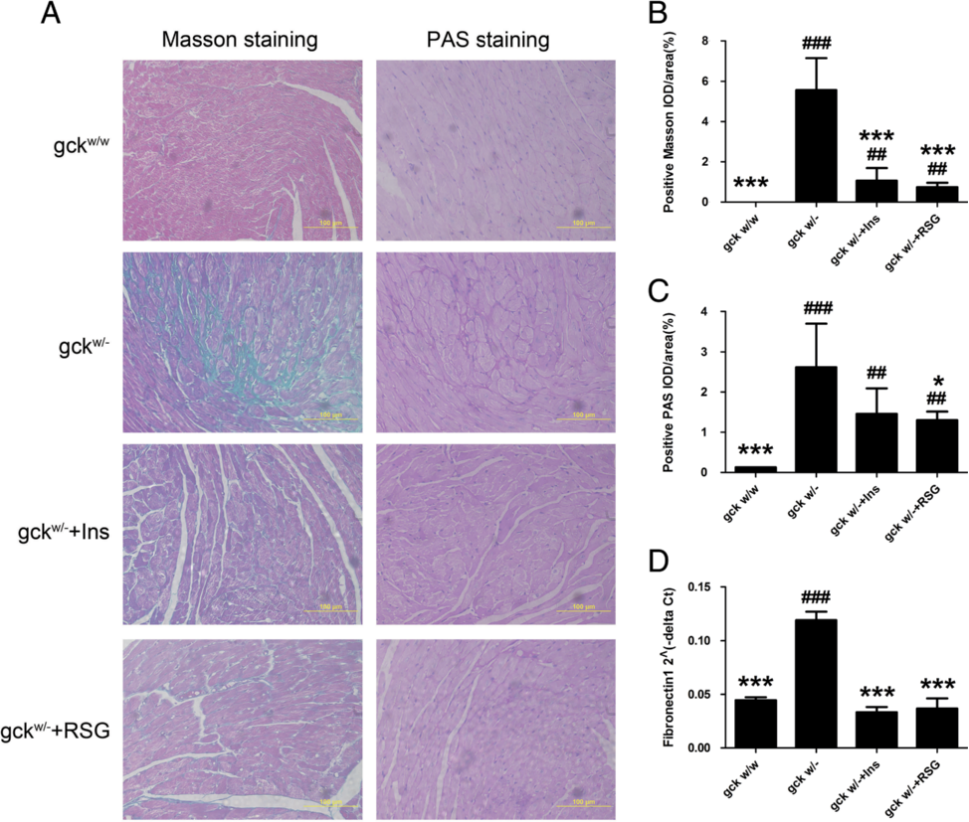
**Cardiac fibrotic levels in 60-week old liver-specific gck knockout mice.** Cardiac fibrosis levels were investigated in wild-type (gck^w/w^) and gck knockout (gck^w/–^) mice as well as knockout mice treated with insulin or rosiglitazone for 4 weeks. Images of PAS and Masson staining are shown in **(A)** with positive Masson IOD/area **(B)**, and positive PAS IOD/area **(C)** shown. The relative amount of mRNA for fibronectin 1 in the myocardial, which was determined by real-time PCR and corrected with beta actin as an internal standard, is shown in **(D)**. n = 3 for all samples. Asterisk (*) refers to statistical significance (P < 0.05 for * and P < 0.001For ***) in comparisons with gck^w/–^ mice, while # refers to statistical significance (P < 0.005 for ## and P < 0.001 for ###) in comparisons with gck^w/w^ mice.

Under the microscope, in gck^w/w^ mice, the distribution of collagen (Masson positive material) was almost uniform, where the collagen fiber network among adjacent cells was intact and had lower levels of collagen fiber content (Figure 5). In the gck^w/–^ mice, significantly increased levels of myocardial collagen, which was broken and arranged in a disordered collagen fiber network around the myocardial cells, was observed (Figure 5). The relative content of collagen or glycoproteins (PAS-positive material) in myocardial tissue was measured and the results showed, compared with gck^w/w^ mice, that the myocardial collagen or glycoproteins content of gck^w/–^ mice was significantly increased (p < 0.01), suggesting the accumulation of collagen and glycoproteins in this mouse model. Compared with gck^w/–^ mice, the collagen and glycoprotein content in the rosiglitazone or insulin treated groups were decreased significantly (p < 0.05), suggesting that both rosiglitazone and insulin inhibit the accumulation of collagen and glycoproteins (Figure 5). As shown in Figure 5D, the levels of fibronectin1 mRNA in the myocardium of the gck^w/–^ mice was significantly higher than in gck^w/w^ mice and both rosiglitazone and insulin restore levels to the wild-type levels (p < 0.05).

### Liver-specific gck gene knockout augments mitochondrial volume density and number

**Figure 6:**
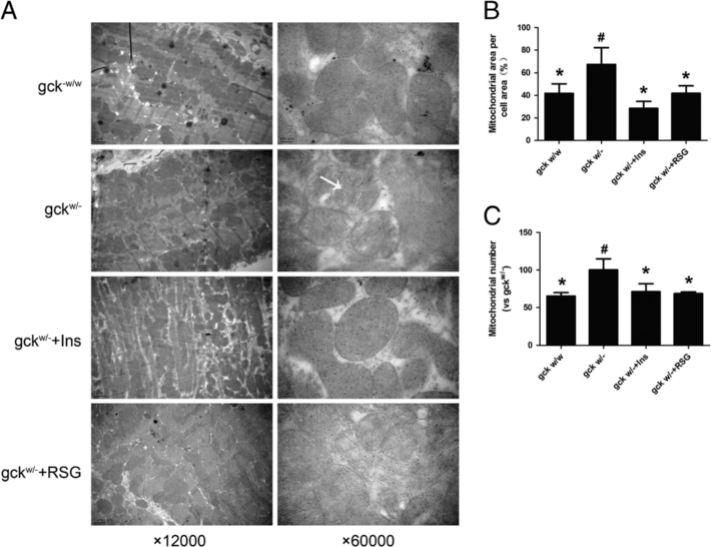
**Changes in mitochondrial ultrastructure in the hearts of 60-week old liver-specific gck knockout mice.** Representative electron micrographs (×12,000 or × 60,000) **(A)**, stereological quantification of mitochondrial volume density **(B)**, and mitochondrial number **(C)** of wild-type (gck^w/w^) and gck knockout (gck^w/–^) mice as well as knockout mice treated with insulin or rosiglitazone for 4 weeks are shown. Cristae density (white arrow) was decreased in the gck^w/–^mice. Asterisk (*) refers to statistical significance (P < 0.05) in comparisons with gck^w/–^ mice, while # refers to comparisons with gck^w/w^ mice.

Mitochondrial structure and myocardial ultrastructure were evaluated by electron microscopy. Cristae density of the mitochondria was decreased in the gck^w/–^ mice (Figure 6A). Mitochondrial volume density (Figure 6B) and number (Figure 6C) were increased in the gck^w/–^ mice, compared to gck^w/w^ mice. These properties were restored to wild-type levels by treatment with rosiglitazone or insulin (p < 0.05).

### Liver-specific gck gene knockout impairs the levels of the insulin receptor and Akt

**Figure 7:**
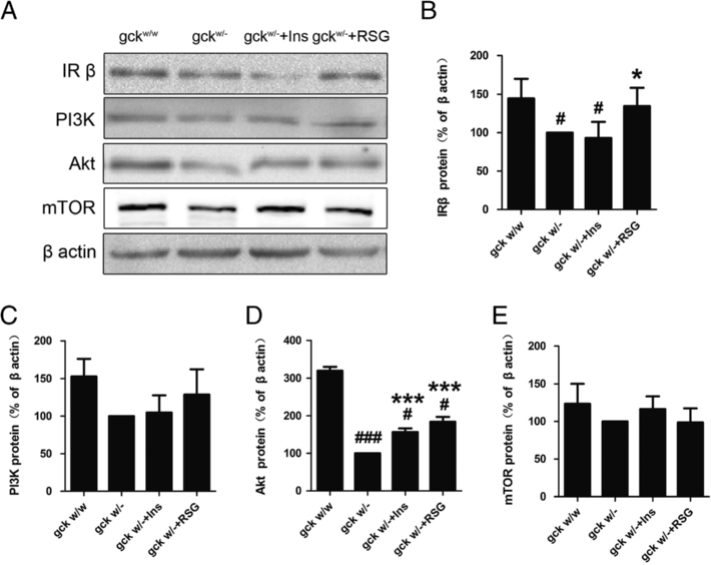
**Changes in the protein levels of Insulin signaling pathway in hearts of 60-week old liver-specific gck knockout mice.** Representative Western blots images **(A)** with quantification of IR **(B)**, PI3K **(C)**, Akt **(D)**, and mTOR **(E)** from heart homogenates of wild-type (gck^w/w^) and gck knockout (gck^w/–^) mice as well as knockout mice treated with insulin or rosiglitazone for 4 weeks are shown. n = 3 for all samples. Asterisk (*) refers to statistical significance (P < 0.05 for * and P < 0.001 for ***) in comparisons with gck^w/–^ mice, while # refers to statistical significance (P < 0.05 for # and P < 0.001 for ###) in comparisons with gck^w/w^ mice.

The insulin signaling pathway plays important roles in the pathogenesis of type 2 diabetes mellitus. Protein levels of the insulin receptor and Akt were significantly down-regulated in the gck^w/–^ mice, compared with gck^w/w^ mice (Figure 7), and this change was reversed by treatment with rosiglitazone (p < 0.05). No significant changes in the levels of PI3K or mTOR proteins were observed (Figure 7). These results indicate that the gck^w/–^ mice show a significant modulation of the insulin signaling pathway, which might be linked to additional deleterious consequences for the gck^w/–^ mice.

### Liver-specific gck gene knockout reduces AMPKβ and ACC phosphorylation

**Figure 8:**
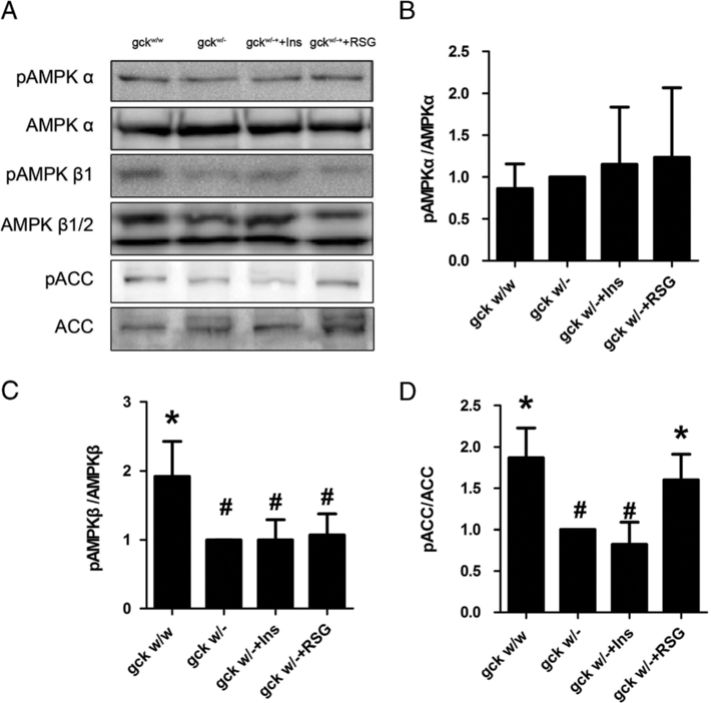
**Levels of phosphorylated AMPK and ACC in the hearts of 60-week old liver-specific gck knockout mice.** Representative Western blot images **(A)** and quantification of the levels of p-AMPKα/AMPKα **(B)**, p-AMPKβ/AMPKβ **(C)**, and p-ACC/ACC **(D)** in heart homogenates of wild-type (gck^w/w^) and gck knockout (gck^w/–^) mice as well as knockout mice treated with insulin or rosiglitazone for 4 weeks are shown. Phosphorylated AMPK and ACC levels were measured by Western blots. n = 3 for all samples. Asterisk (*) refers to statistical significance (P < 0.05) in comparisons with gck^w/–^ mice, while # refers to comparisons with gck^w/w^ mice.

Protein levels of p-AMPKβ1were significantly down regulated in the gck^w/–^ mice, compared with gck^w/w^ mice (p < 0.05, Figure 8). In addition, rosiglitazone and insulin did not affect AMPKβ phosphorylation. p-AMPKα protein levels did not change significantly when the gck^w/–^ mice were treated with rosiglitazone or insulin. To examine whether AMPK regulates ACC phosphorylation in gck^w/–^ mice, we examined the protein levels of p-ACC. Surprisingly, the level of p-ACC was dramatically lower in the gck^w/–^ mice in comparison to gck^w/w^ mice (p < 0.05). The level of p-ACC was restored to wild-type levels by treatment with rosiglitazone (p < 0.05), but not with insulin (Figure 8).

## Discussion

MODY2 in humans is characterized by mild but chronic hyperglycemia, with some MODY2 patients displaying peripheral insulin resistance, but diabetes-associated macro- and micro-vascular complications, overweight, and dyslipidemia are uncommon [22,23]. Patients can usually manage their blood glucose levels by diet and exercise alone [11]. The liver-specific gck knockout mice (gck^w/–^) appear normal at birth, with measurement of plasma insulin, triglyceride, cholesterol, ALT, and AST showing no difference compared with wild-type mice at up to 6 weeks of age [15]. These parameter are similar to those observed in MODY2 patients. Fasting blood glucose levels in these mice increase with age and these mice spontaneously develop hyperglycemia after 6 weeks of age with impaired glucose tolerance and a decreased accumulation of hepatic glycogen [15]. These mice however, do not exhibit signs of obesity with aging [15]. No evidence of autoimmunity in the pancreatic islets is exhibited by these knockout mice [15]. The gck^w/–^ mouse, thus, may serve as a model of human MODY2 diabetes as it is associated with mild hyperglycemia and insulin resistance without hypoinsulinemia. These mice (gck^w/–^ mice) were used to investigate the functional and structural changes in the myocardium that result from long-term (60 weeks) reduced liver gck expression (yielding mild hyperglycemia) followed by 4 weeks with or without treatment with insulin or rosiglitazone.

Diabetic cardiomyopathy describes diabetes-associated changes in myocardial structure and function. HOMA-IR is based on the dynamic interaction between glucose and insulin output and is a well-documented clinical representation of insulin resistance when compared to the gold standard reference, the euglycemic clamp [24]. In several studies, an association between diabetic cardiomyopathy and HOMA-IR has been found. The HOMA index is an independent determinant of LV diastolic function [25]. Insulin resistance has been correlated with increased left ventricular mass and risk of heart failure [26]. In this study, we found that in gck^w/–^ mice both serum glucose levels and insulin resistance were dramatically elevated compared with the control group (gck^w/w^ mice). It has been demonstrated that the impairment of glucose homeostasis is able to affect the severity of heart disease.

MLC2 is part of the myosin complex, a hexameric complex of two heavy chains and four light chains, predominantly expressed in cardiac ventricle muscle [21]. The regulatory light chains of the myosin complex, MLC2, can be phosphorylated leading to a conformational change, which consequently affects muscle contraction [27]. Mutations in the human MLC2 gene have been associated with hypertrophic cardiomyopathy and lack of MLC2 in mice is embryonic lethal due to cardiac dysfunction that results in heart failure, a massive cardiac enlargement, wall thinning, and abnormalities in myofibril assembly [21]. In this study, we found that in gck^w/–^ mice the level of MLC2 protein expression was dramatically elevated compared with the control group (gck^w/w^ mice). Koka et al. [28] revealed a coordinated down regulation of cytoskeletal contractile proteins such as myosin heavy-chainα (αMHC), and an up-regulation of MLC2 in diabetic mice, a result consistent with our results. The specific mechanism underlying these changes is unclear and we hypothesized that an increase in MLC2 levels may lead to myocardial hypertrophy by interfering with the assembly of myofibrils.

In several studies, an association between diabetic cardiomyopathy and cardiac hypertrophy, increased myocardial stiffness and reduced chamber compliance has been found [29]. Clinical studies have demonstrated that diabetic cardiomyopathy is manifested with left ventricular hypertrophy associated with systolic/diastolic dysfunction and cardiac fibrosis in diabetic patients [30]. In the present study, we observed cardiac hypertrophy in the gck^w/–^ mouse, which was observed as an increase in the left ventricular wall thickness, a decrease of the left ventricular internal dimension, longer PR and QRS intervals, and increased myocardium MLC2 protein levels. Treatment with rosiglitazone results in PR and QRS intervals (Table 3), and MLC2 protein levels (Figure 3) being restored to wild-type levels, however, LVPW (Table 2) worsened after treatment with insulin or rosiglitazone.

A significant increase in collagen deposition around the intramural cardiacvessels and between myofibrils in heart biopsies from diabetic patients has been found [31]. The PAS reaction is due to the accumulation of glycoprotein in the myocardium, which leads to interstitial fibrosis. Formation of glycoprotein has been suggested to be due to the increased formation of glucose-derived aldehydes, giving rise to so-called advanced glycation end products (AGEs) [32]. The accumulation of glycoprotein increases myocardial stiffness by cross-linking. As seen in our study, reduced gck expression in the liver had an unfavorable effect on cardiac morphology and lead to the accumulation of collagen (Masson positive material) and glycoproteins (PAS positive material) in the myocardium (Figure 5). Glycoproteins increase intracellular oxidative stress, which contributes to additional cell damage. Oxidative stress is associated with mitochondrial damage and plays an important role in the development of diabetic cardiomyopathy [33].

Hyperglycemia-induced impairment of redox balance is considered to be a key trigger of diabetic complications, through the up-regulated generation of reactive oxygen species (ROS) [34], together with an impaired ability of the endogenous antioxidant defense system to remove them [35]. The heart is particularly susceptible to oxidative damage, as it possesses lower levels of endogenous antioxidants in comparison to other organs. Cyba and Cybb are essential subunits of the NADPH oxidase, an enzyme considered to be a major source of O_2_˙^-^ in cardiac cells catalyzing the one-electron reduction of oxygen using NADH or NADPH as the electron donor [36]. Recent evidence suggests that NADPH oxidase derived superoxide is a key trigger of mitochondrial dysfunction and up regulated mitochondrial superoxide generation [37]. SOD is one of the most important physiological antioxidants against free radicals and functions to prevent subsequent lipid peroxidation. Lipid peroxidation, which is a normal phenomenon that occurs continuously at lowlevels in every individual, is the most common consequence of oxidative stress. MDA is the end product of lipid peroxidation and is toxic to cells and cell membranes. Hyperglycemia contributes to the activation of free radicals and leads to tissue damage in different organs, such as endothelial dysfunction, hypertrophy and fibrosis [38]. In the present study, we found that the MDA content and Cyba mRNA expression levels were significantly higher in gck^w/–^ mice than in gck^w/w^ control mice, which is concordant with the finding of significantly decreased SOD activity in the gck^w/–^ mice. These results suggest that reduced liver gck expression may lead to a decreasein the antioxidant capacity of the diabetic myocardium, contributing significantly to oxidative stress and the resulting myocardial damage.

At the same time, our study showed that the mitochondrial volume density and number were increased in the gck^w/–^ mice compared to gck^w/w^ mice (Figure 6). After treatment with rosiglitazone or insulin, these parameters were restored to more normal values. Mitochondria are the center of fatty acid and glucose metabolism and thus are highly likely to be impacted by impaired metabolism associated with diabetes. Increased number and size of >mitochondria might be an adaptive response to hyperglycemia [39]. It has recently been shown that insulin resistance drives the cardiac mitochondrial biogenesis regulatory program via PPARα, where activation was increased due to increased fat uptake and oxidation [40,41]. The liver-specific glucokinase knockout mouse experienced long-term hyperglycemia, which results in decreased insulin receptor levels and ultimately leading to insulin resistance and attenuated glucose uptake [42]. Insulin resistance drives the cardiac mitochondrial biogenesis regulatory program, which lead to increased mitochondrial volume density and number [43]. Increased oxidative phosphorlyation and impairment in the electron transport chain contribute to increased ROS and superoxide (O_2_˙^-^) production [44]. These changes cause morphological and functional abnormalities in cardiac tissue, thus suggesting that oxidative stress might be the unifying factor for the damaging effects of hyperglycemia [45].

The molecular signaling pathways implicated in myocardial damage in gck^w/–^ mice have not been fully resolved. We demonstrate that the cardiac insulin receptor and Akt protein levels, key factors of the IR-PI3K-Akt pathway, were reduced in gck^w/–^ mice, but were restored to wild-type levels by insulin or rosiglitazone treatment. The IR-PI3K-Akt pathway induces a hypertrophic response associated with cardioprotection [46], in which cardiac structure and function are preserved. Based on our observations, we postulate that prolonged exposure to increased levels of serum glucose depresses protectiveIR-PI3K-Akt signaling. Preservation of insulin receptor and Akt levels in the rosiglitazone-treated diabetic myocardium might thereby confer protection against pathological cardiac hypertrophy.

Metabolic disorders play major roles in the pathogenesis of diabetic cardiomyopathy. AMPK is a central regulator for glucose and fatty acid metabolism in mammalian cells, which acts as anenergy sensor, responding to a rise in AMP levels by increasing ATP generating pathways and reducing ATP-consuming pathways [47]. AMPK is a heterotrimeric complex composed of a catalytic α subunit and regulatory β and γ subunits. Phosphorylation at Ser108 of theβ1 subunit seems to be required for the activation of AMPKenzyme. AMPK phosphorylation inhibits fatty acid and cholesterol synthesis and gluconeogenesis in the liver and stimulates fatty acid uptake and oxidation, glucose uptake, and mitochondrial biogenesis in skeletal muscle [48].

ACC is a cytosolic enzyme that catalyzes the carboxylation of cytosolic acetyl-CoA to form malonyl-CoA, which is the pivotal step of the fatty acid synthesis pathway [49]. ACCβ is the major isoform in heart. We demonstrated that the levels of phosphorylated AMPK and ACC were dramatically lower in gck^w/–^ mice, but only p-ACC was restored to wild-type levels with rosiglitazone treatment. It has previously been reported that AMPK regulates ACC phosphorylation [49]. Phosphorylation by AMPK inhibits the enzymatic activity of ACC, and in turn malonyl-CoA levels [50]. These results suggest that the fatty acid synthesis pathway may be enhanced in the myocardium of gck^w/–^ mice, due to a reduction in ACC phosphorylation. The accumulation of fatty acid also causes a pathological ROS accumulation, which leads to damage in cardiomyocytes. It has been hypothesized that the dysregulation of the AMPK/ACC fuel-sensing and signaling network is a key factor in the development of insulin resistance and cardiac hypertrophy [51].

## Conclusions

**Figure 9:**
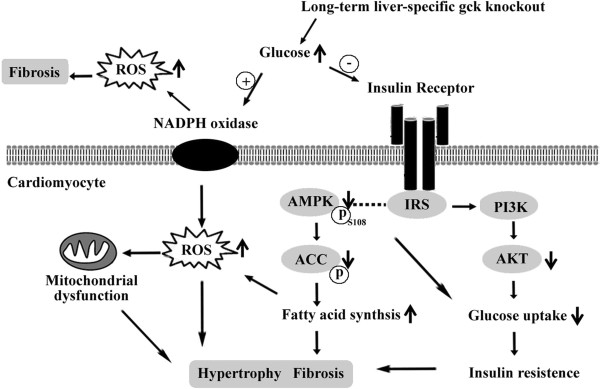
**The influence of liver-specific glucokinase knockout on the myocardium.** The liver-specific glucokinase knockout mouse experiences long-term hyperglycemia, which induced decreased levels of insulin receptor. Disrupting the early signaling events in the insulin pathway also has downstream effects on other proteins including Akt and AMPK, ultimately leading to insulin resistance and attenuated glucose uptake. Fatty acid synthesis increased in cardiomyocyte by decreased AMPK phosphorylation and subsequent increased ACC activity. Increased glucose stimulates NADPH oxidase expression. NADPH oxidase-derived superoxide generation then contributes to mitochondrial dysfunction, leading to a further increase in superoxide generation. Insulin resistance, increasing fatty acid synthesis and oxidative stress induced cardiomyocyte hypertrophy and fibrosis.

In conclusion, for the first time, the present study has established a diabetic cardiomyopathy model using a liver-specific glucokinase knockout mouse model. The liver-specific glucokinase knockout mouse experiences long-term hyperglycemia, which induces decreased levels of insulin receptor. Disrupting the early signaling events in the insulin pathway also has downstream effects on other proteins including Akt and AMPK, ultimately leading to insulin resistance and attenuated glucose uptake. Fatty acid synthesis increased in cardiomyocyte by decreased AMPK phosphorylation and subsequent increased ACC activity. Increased glucose stimulates NADPH oxidase expression. NADPH oxidase-derived superoxide generation then contributes to mitochondrial dysfunction, leading to a further increase in superoxide generation. Insulin resistance, increasing fatty acid synthesis and oxidative stress induced cardiomyocyte hypertrophy and fibrosis (Figure 9). Rosiglitazone treatment may partly protect against diabetic cardiomyopathy by modulating cardiac lipid metabolism, oxidative stress and altering the expression of a set of proteins involved in cardiac damage, but that myocardial structural and functional changes cannot be full-reversed in gck^w/–^ mice. These findings suggest that the gck^w/–^ mice may be utilized as an effective model for research on diabetic cardiomyopathy. Reduced gck expression in the liver may induce diabetic cardiomyopathy by up regulating NADPH oxidase and down regulating insulin receptor and p-AMPK protein levels.

## Abbreviations

DCM: Diabetic cardiomyopathy; DM: Diabetes mellitus; MODY: Maturity-onset diabetes of the young; gck: Glucokinase; PPARγ: Peroxisome proliferator activated receptor γ; ipGTT: Intraperitoneal glucose tolerance test; HOMA: Homeostasis model assessment; EF: Ejection fraction; FS: Fractional shortening; ECG: Electrocardiogram; MLC2: Myocardium myosin light chain; AMPK: Adenosine 5'-monophosphate (AMP)-activated protein kinase; ACC: Acetyl-CoA carboxylase; SOD: Superoxide dismutase; MDA: Malondialdehyde; LVID;d: Left ventricle internal dimension during diastole; LVID;s: Left ventricle internal dimension during systole; LVPW;d: Left ventricle posterior wall thickness during diastole; LVPW;s: Left ventricle posterior wall thickness during systole; Cyba: Cytochrome b-245 alpha; Cybb: Cytochrome b-245 beta.

## Competing interests

The authors declare that they have no competing interests.

## Authors’ contributions

HT and GN are the guarantors of this work, and as such, they had full access to all the data in this study and take full responsibility for the integrity of the data and accuracy of the data analysis. HL researched data and wrote the manuscript; DMI reviewed and edited the manuscript; XW, YM, RH, WX, ZL, NZ, LJ and TG researched data; ZL reviewed the manuscript. All authors read and approved the final manuscript.
